# Fermented Sipjeondaebo-tang Alleviates Memory Deficits and Loss of Hippocampal Neurogenesis in Scopolamine-induced Amnesia in Mice

**DOI:** 10.1038/srep22405

**Published:** 2016-03-04

**Authors:** Hee Ra Park, Heeeun Lee, Hwayong Park, Won-Kyung Cho, Jin Yeul Ma

**Affiliations:** 1 Korean Medicine (KM)-Application Center, Korea Institute of Oriental Medicine (KIOM), Daegu, Republic of Korea

## Abstract

We investigated the anti-amnesic effects of SJ and fermented SJ (FSJ) on scopolamine (SCO)-induced amnesia mouse model. Mice were orally co-treated with SJ or FSJ (125, 250, and 500 mg/kg) and SCO (1 mg/kg), which was injected intraperitoneally for 14 days. SCO decreased the step-through latency and prolonged latency time to find the hidden platform in the passive avoidance test and Morris water maze test, respectively, and both SCO effects were ameliorated by FSJ treatment. FSJ was discovered to promote hippocampal neurogenesis during SCO treatment by increasing proliferation and survival of BrdU-positive cells, immature/mature neurons. In the hippocampus of SCO, oxidative stress and the activity of acetylcholinesterase were elevated, whereas the levels of acetylcholine and choline acetyltransferase were diminished; however, all of these alterations were attenuated by FSJ-treatment. The alterations in brain-derived neurotrophic factor, phosphorylated cAMP response element-binding protein, and phosphorylated Akt that occurred following SCO treatment were protected by FSJ administration. Therefore, our findings are the first to suggest that FSJ may be a promising therapeutic drug for the treatment of amnesia and aging-related or neurodegenerative disease-related memory impairment. Furthermore, the molecular mechanism by which FSJ exerts its effects may involve modulation of the cholinergic system and BDNF/CREB/Akt pathway.

In adult hippocampal neurogenesis, the newly-generated neural progenitor cells (NPCs) in the dentate gyrus (DG) of the hippocampus become new neurons and are functionally integrated into the existing hippocampal neuronal circuit. The hippocampal neuronal circuit is closely associated with cognitive functioning, including learning, memory, retention, and restoration^1^,^2^. However, aging-related dementia and neurodegenerative disorders, including Alzheimer’s disease (AD), are irreversible and are progressive diseases characterized by hippocampal neurogenesis impairments, deficits in cognitive function, and difficulties remembering newly acquired information^3^,^4^. The normal cholinergic system in the brain affects hippocampal neurogenesis and cognitive function by modulating neurogenic mechanisms such as those involving brain-derived neurotrophic factor (BDNF) and cAMP response element-binding protein (CREB)^5^. Impairments in learning and memory that are associated with AD and aging have been attributed primarily to cholinergic dysfunction, including impaired acetylcholine (ACh) release and increased acetylcholinesterase (AChE) activity in the neurons of the central nervous system (CNS)^6^,^7^. Therefore, current therapeutic drugs that are based on the cholinergic system, such as AChE inhibitors (donepezil, galantamine), have been evaluated for treatment of AD and dementia.

Sipjeondaebo-tang (SJ; Shi Quan Da Bu Tang in Chinese and Juzentaihoto in Japanese) is a widely used traditional herbal medicine in East Asia that is composed of 12 natural herbs. SJ has been used for the treatment of reduced vitality, fatigue, and asthenia. Previous papers have reported that SJ possesses various biological properties, including anti-inflammatory properties, anti-cancer activity, gastroprotective effects and immune cell activation^8^,^9^,^10^,^11^. Recent studies using the AD animal model have suggested that the neuroprotective effects of SJ are due to reduced Aβ aggregation and reduced activation of microglia^12^,^13^. However, the effects of SJ on hippocampal neurogenesis and cognitive functioning have not been studied. Our previous study showed that SJ fermented by *Lactobacillus* (FSJ) was associated with increased anti-inflammatory activities on lipopolysaccharides (LPS)-stimulated RAW 264.7 cells compared with SJ^14^. Interestingly, several studies have indicated that fermentation of natural herbs with *Lactobacillus* strengthen active components, increase physiological potency, and improve absorption efficiency^15^,^16^. Therefore, in this study, we investigated the potential anti-amnesic effects of SJ and FSJ on hippocampal neurogenesis and cognitive functioning in the scopolamine (SCO)-induced amnesia mouse model. Additionally, we investigated the BDNF, CREB, and Akt signaling pathways of SJ and FSJ in SCO-induced amnesic mice.

## Results

### FSJ improved SCO-induced step-through latency impairments in the passive avoidance test

**Figure 1:**
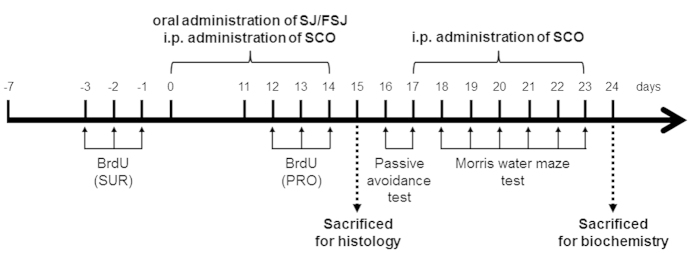
Schematic overview of the *in vivo* experimental procedure. C57BL/6 mice (5-week-old) were administered CON, SCO (1 mg/kg), SJ, or FSJ (125, 250, or 500 mg/kg) for 14 days. To assess newborn cell proliferation (PRO), BrdU (50 mg/kg) injections were given on the final 3 days (day 12–14) of drug administration. For newborn cell survival assessments (SUR), BrdU was injected on the 3 days prior to drug administration. Mice were intracardially perfused on day 15 and brain tissue sections were processed for immunohistochemistry staining. Behavioral tests (passive avoidance test and Morris water maze test) were performed on days 16–23 and mice were sacrificed on day 24 for biochemical studies.

**Figure 2:**
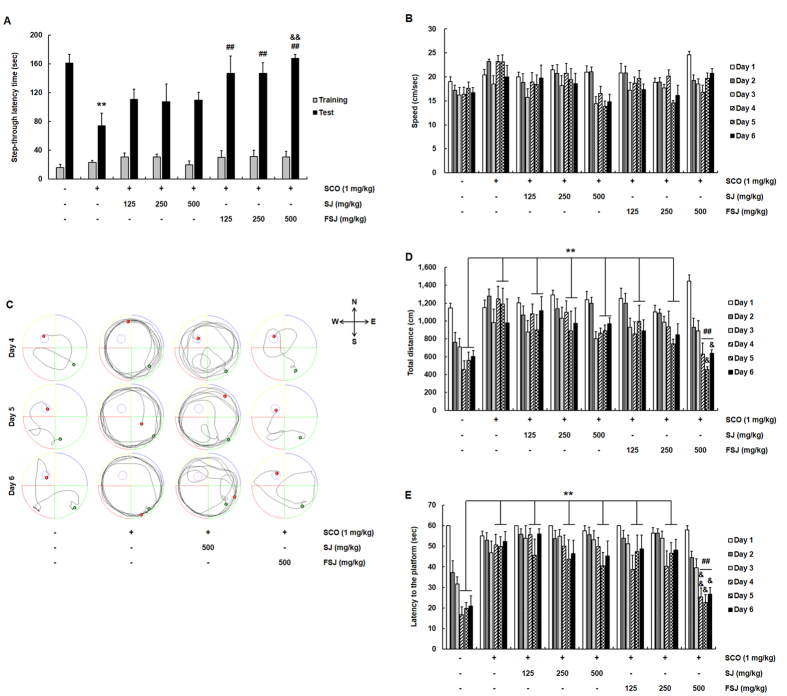
Effects of SJ and FSJ on memory retention and spatial memory in the SCO-induced mouse model of cognitive impairment. (**A**) In the passive avoidance test, SCO (1 mg/kg) or the same volume of saline (CON group) was injected in mice 30 min prior to the test. The latency time to cross from the light to dark compartment was measured during training and on test days. Data are presented as the mean ± SEM (*n* = 6–8 mice/group). ^**^p < 0.01, compared with CON-treated group; ^##^p < 0.01, compared with SCO-treated group; ^##^p < 0.01, compared with 500 mg/kg SJ-treated group. In (**B**–**E**), the MWM task was performed to determine spatial reference learning and memory in SCO-induced memory deficit mice that were treated with either SJ or FSJ. SCO (1 mg/kg) or the same volume of saline (CON group) was injected in mice 30 min prior to the MWM experiments. Changes in the swimming speed (in **B**) and total distance moved (in **D**) during the 6 test days are shown. (**C**) Representative swimming paths of mice in each treatment group at days 4–6 of the spatial reference trial test are shown. (**E**) The escape latency time to reach the hidden platform during the 6 days of testing is shown. Data are presented as the mean ± SEM (*n* = 6–8 mice/group). *p < 0.05, **p < 0.01, compared with CON-treated group; ^#^p < 0.05, ^##^p < 0.01, compared with SCO-treated group; ^&^p < 0.05, ^&&^p < 0.01, compared with 500 mg/kg SJ-treated group.

SCO is a nonselective muscarinic receptor antagonist associated with learning and memory deficits observed in the aging-related and dementia-related symptoms of cognitive impairment^17^. Thus, we used the SCO-induced amnesia mouse model to evaluate the potential anti-amnesic effects of SJ or FSJ administration^18^. To investigate the effects of SJ and FSJ on the brain, 5-week-old C57BL/6 male mice were orally administered 0.9% physiological saline (CON group; vehicle-control; and SCO group) or 0.9% physiological saline containing SJ or FSJ at 2 h after SCO injection for 14 days. With the exception of the CON group, SCO was injected once intraperitoneally (i.p.) to mice 30 min before the behavior test (Fig. 1). First, to explore the acute toxicity of SJ and FSJ administration, body weight was measured every 4 days. In each animal group, body weight was observed to increase normally, and no significant differences were observed between groups (see Supplemental Fig. S1). Additionally, we measured body weight and the weight of organs such as the heart, lungs, liver, kidneys, and spleen. The results showed that administration of 500 mg/kg of SJ or FSJ for 14 days had no effect on body or organ weights (see Supplemental Fig. S1). Furthermore, abnormal external appearances, gross lesions in internal organs, and peculiar behaviors were not observed. These results indicated that continuous oral administration of SJ or FSJ at a dose of 500 mg/kg for 14 days did not cause any toxicity to mice. In a previous paper investigating the safety of SJ for human consumption, SJ was orally administered to male and female SD rats at a dose of 2,000 mg/kg/day for 13 weeks, and no adverse effects on body or organs weights were observed^19^. To assess the effects of SJ and FSJ on hippocampus-dependent contextual-fear memory, experimental animals performed the step-through latency passive avoidance test. At training day (day 0), mice received an electric foot shock, and the step-through latency time did not significantly differ among the groups (Fig. 2A; p > 0.9999). The memory retention test was performed 24 h after training. SCO-treated mice showed a reduced step-through latency time compared with CON-treated mice (p < 0.0001), indicating that SCO treatment of mice reliably induces amnesia in treated animals. Compared with the SCO-treated mice, latency time was similarly reduced in the 125, 250, and 500 mg/kg SJ-treated mice (p > 0.9999, p > 0.9999, p = 0.9087, respectively). However, 125, 250 and 500 mg/kg of FSJ significantly increased the step-through latency time in the passive avoidance test (p = 0.0019, p = 0.0020, p = 0 < 0.0001, respectively), indicating that FSJ significantly improved memory retention. Interestingly, latency time for the 500 mg/kg FSJ-treated mice was higher than that for the 500 mg/kg SJ-treated mice (p = 0.0002). Moreover, two-way ANOVA analysis showed that there was a significant difference in effectiveness when comparing between treatment × time interaction effects in the passive avoidance test (treatment effect [F_(7,90)_ = 4.253, p = 0.0004], time effect [F_(1,90)_ = 290.1, p < 0.0001], and treatment × time interaction [F_(7,90)_ = 3.735, p = 0.0014]).

### FSJ ameliorated SCO-induced learning and memory deficits in the Morris water maze (MWM) test

The MWM test was used to evaluate neurocognitive functions associated with learning and memory. To determine the effects of SJ and FSJ on hippocampus-dependent learning and memory, we tested reference learning and memory performance in the MWM test. Two-way ANOVA for swim speed revealed that there were no statistical differences between the treatment × time interaction effects in the MWM test (Fig. 2B; treatment effect [F_(7,257)_ = 6.117, p < 0.0001], time effect [F_(5,257)_ = 6.292, p < 0.0001], and treatment × time interaction [F_(35,257)_ = 1.236, p = 0.1797]). Swim speed did not differ between groups, indicating that administration of CON, SCO, SJ, or FSJ did not affect the movement or motor activity of mice. As shown in Fig. 2C, the escape route of CON-treated mice was short and direct; however, in SCO-treated mice, the swimming pattern was confined more to side walls rather than to the center of the pool as observed in CON-treated mice. Similar to the SCO-treated mice, the 500 mg/kg SJ-treated mice also swam in a circular pattern and did not find the hidden platform. However, the swim pattern of the 500 mg/kg FSJ-treated mice was better than that in the SCO-treated mice and SJ-treated mice, indicating better learning and memory. These results indicate that SCO-induced learning and memory deficits were improved in mice administered FSJ but not SJ. Figure 2D shows the total moved distance to the platform during the 6 days of testing. On testing days 4–6, in comparison to CON-treated mice, the total moved distance was significantly greater in SCO-treated mice and mice that received treatment doses of either 125 or 250 mg/kg of either SJ or FSJ (p > 0.9999). The total moved distance of the 500 mg/kg FSJ-treated mice on testing days 4–6 was lower than that for the SCO-treated mice (p < 0.001) but not lower than that for mice treated with SJ at the 500 mg/kg dose (p > 0.9999). Interestingly, latency times for the 500 mg/kg FSJ-treated mice were lower than those for the 500 mg/kg SJ-treated mice on days 5 and 6 (p < 0.0001, p = 0.0012, respectively). Moreover, there were significant differences between the treatment × time interaction effects of the total moved distance in the MWM test on the 6 days (Fig. 2D; treatment effect [F_(7,257)_ = 9.164, p < 0.0001], time effect [F_(5,257)_ = 14.33, p < 0.0001], and treatment × time interaction [F_(35,257)_ = 1.482, p = 0.0462]). SCO-treated mice exhibited a significantly higher latency time to reach the hidden platform on days 4, 5, and 6 compared to CON-treated mice (Fig. 2E; p < 0.0001, p = 0.0006, p = 0.0002, respectively). However, 500 mg/kg of FSJ significantly reduced the latency time to reach the hidden platform on days 4, 5, and 6 compared to SCO-treated mice (Fig. 2E; p = 0.0035, p = 0.0010, p = 0.0026, respectively). Interestingly, latency times for the 500 mg/kg FSJ-treated mice were lower than the 500 mg/kg SJ-treated mice on days 4, 5 and 6 (p = 0.0017, p = 0.0348, p = 0.0397, respectively). The interaction analysis showed that the treatment × time interaction was affected in the MWM test (Fig. 2E; treatment effect [F_(7,257)_ = 19.12, p < 0.0001], time effect [F_(5,257)_ = 17.63, p < 0.0001], and treatment × time interaction [F_(35,257)_ = 1.515, p = 0.0376]). Taken together, these results indicate that FSJ but not SJ can prevent SCO-induced neurocognitive deficits related to memory retention and spatial memory.

### Effects of SJ and FSJ on proliferation and survival of newly-generated cells in the hippocampus of SCO-induced amnesia in mice

**Figure 3:**
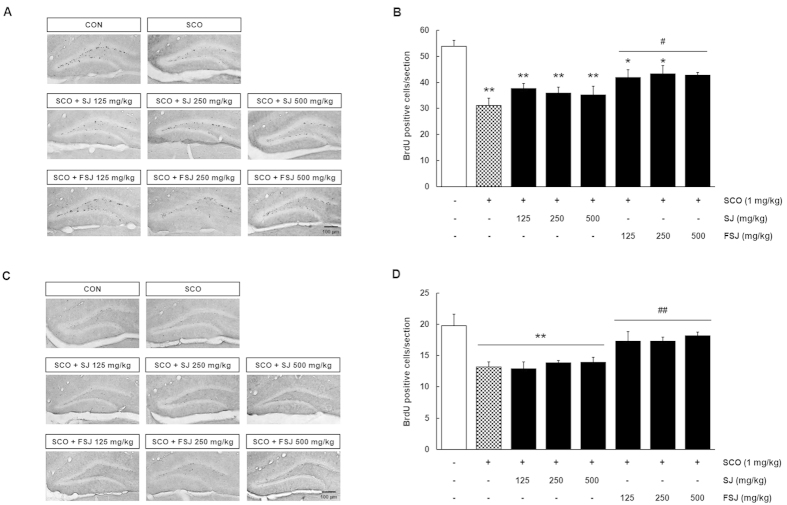
The SCO-induced loss of proliferation and survival of newly-generated BrdU-positive cells in the DG of the hippocampus was prevented by FSJ. (**A**) Representative images indicating proliferation of BrdU-positive cells in the DG of each experimental group are shown. In the proliferation study, newly-generated cells existed in the SGZ of the DG. The scale bar = 100 μm. (**B**) The graph shows the number of BrdU-positive cells in the proliferation study. Data are presented as the mean ± SEM (*n* = 5 mice/group). **p < 0.01, compared with CON-treated group; ^##^p < 0.01, compared with SCO-treated group. (**C**) Representative images indicating survival of BrdU-positive cells in the DG of each experimental group are shown. Surviving newborn cells migrated into the GCL of the DG. The scale bar = 100 μm. (**D**) The graph shows the number of BrdU-positive cells in the survival study. Data are presented as the mean ± SEM (*n* = 5 mice/group). **p < 0.01, compared with CON-treated group; ^##^p < 0.01, compared with SCO-treated group.

To determine the effects of SJ and FSJ on cell proliferation and survival in the hippocampus, 5-week-old C57BL/6 mice were administered either SJ or FSJ (125, 250 or 500 mg/kg, orally) and SCO (1 mg/kg, i.p) for 14 days (Fig. 1). In addition, BrdU was injected for 3 days after (in the proliferation study) or before (in the survival study) administration of each drug (Fig. 1). We used an anti-BrdU antibody to detect proliferative cells in the DG of the hippocampus. In all the proliferation studies, BrdU-positive cells were abundant in the subgranular zone (SGZ) of the DG (Fig. 3A). Administration of SCO reduced the number of newly-generated cells compared with the CON group (Fig. 3A,B; p < 0.0001). The number of BrdU-positive cells in the SJ treatment group slightly increased, although this increase was not significant compared with the SCO group (SJ 125 mg/kg, SJ 250 mg/kg, SJ 500 mg/kg; p = 0.4312, p = 0.6804, p = 0.8361, respectively). The number of newly-generated cells was significantly increased with administration of FSJ (FSJ 125 mg/kg, FSJ 250 mg/kg, FSJ 500 mg/kg; p = 0.0412, p = 0.0174, p = 0.0259, respectively). In the survival study, BrdU-immunoreactive cells were observed in the GCL (Fig. 3C) because newly-generated cells in the SGZ had migrated into the GCL for 14 days. SCO reduced the number of BrdU-positive cells compared with the CON group (Fig. 3C,D; p = 0.0011). Treatment with FSJ significantly increased the survival of newly-generated cells (FSJ 125 mg/kg, FSJ 250 mg/kg, FSJ 500 mg/kg; p = 0.0098, p = 0.0073, p = 0.0090, respectively); however, the number of surviving BrdU-positive cells in the SJ-treatment group did not change (p = 0.999). A one-way ANOVA revealed that proliferation and survival of newly-generated cells was significantly increased in FSJ-treated mice in comparison to the SCO group ([F_(3,13)_ = 3.857, p = 0.0356]; [F_(3,15)_ = 4.850, p = 0.0149], respectively); however, significant differences in cell proliferation and survival were not observed when comparing the SCO-treated and SJ-treated mouse groups ([F_(3,13)_ = 1.042, p = 0.4069]; [F_(3,16)_ = 0.3664, p = 0.7782]). These results indicate that only FSJ prevents the reduction of proliferation and survival of newly-generated cells in the SCO-induced mouse model of amnesia.

### FSJ prevented impairments in hippocampal neurogenesis during SCO treatment

**Figure 4:**
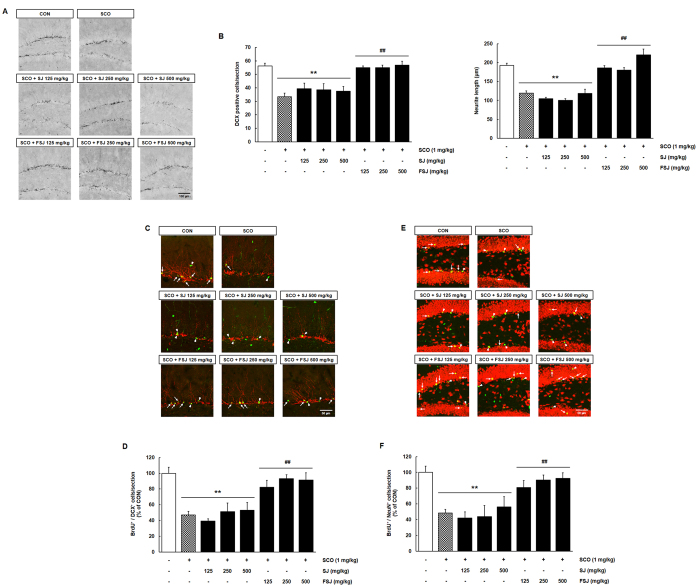
The suppressed hippocampal neurogenesis induced by SCO treatment was restored by treatment with FSJ but not SJ. (**A**) Representative images of DAB immunostaining for DCX in the hippocampal DG of each 14 day drug administration are shown. DCX-positive cells were highly expressed in the nucleus and neurites in the DG. The scale bar = 100 μm. (**B**) The graph shows the number and neurite length of DCX-positive cells in the DG. Data are presented as the mean ± SEM (*n* = 5 mice/group). **p < 0.01, compared with CON-treated group; ^##^p < 0.01, compared with SCO-treated group. In C and E, representative confocal z-stack images of co-localization of BrdU (green) with DCX (in **C**, red) or NeuN (in **E**, red) in brain sections of the survival group are shown. Arrows indicate double positive cells (BrdU^+^/DCX^+^ or BrdU^+^/NeuN^+^). Arrowheads indicate only BrdU^+^ cells. The scale bar = 50 μm. In (**D,F**) quantitative graphs show the percentage of double-labeled positive cells (BrdU^+^/DCX^+^ (in **D**) or BrdU^+^/NeuN^+^ (in **F**)). Data are presented as the mean ± SEM (*n* = 5 mice/group). **p < 0.01, compared with CON-treated group; ^##^p < 0.01, compared with SCO-treated group.

The mossy fibers that belong to newly generated and differentiated neurons grow in a polarized form and reach dendrites in the CA3 region and molecular layer (ML) within approximately 2 weeks^20^. To further investigate the effects of neuronal differentiation in SJ-treated or FSJ-treated mice, we examined brain sections that were stained against DCX, a microtubule-associated protein expressed by neuroblasts and migrating immature neurons, using immunohistochemistry. In all groups, DCX immunoreactivity was observed in nuclei, neurites and branching dendritic trees of cells in the DG (Fig. 4A; [F_(7,32)_ = 11.51, p < 0.0001]; Fig. 4B; [F_(7,32)_ = 34.76, p < 0.0001]). DCX-positive nuclei, branching neurites and cells were markedly reduced in the SCO-treated group compared to the CON group (Fig. 4A,B; p < 0.0001). No differences in DCX immunostaining were observed when comparing between the SJ-treated and SCO-treated groups (p > 0.999), indicating that the SCO-induced loss of neuronal differentiation was not restored with administration of SJ. However, in the FSJ-treated group, the DCX-stained nuclei and branching neurites showed recovery from the SCO-induced decline in neuronal differentiation (FSJ 125 mg/kg, FSJ 250 mg/kg, FSJ 500 mg/kg; p < 0.0001, p = 0.0001, p < 0.0001, respectively), but this effect was not dose-dependent.

During the 2–4 weeks after birth, the newly generated cells in the SGZ of the DG differentiate into mature neurons and migrate to the GCL where they become functionally incorporated into the neuronal circuitry of the DG^1^,^21^. We evaluated neurogenesis using double immunostaining against BrdU and DCX or NeuN, which is expressed in mature neuronal nuclei. Confocal z-stack images and quantitative graphs indicated that SCO-treated mice showed significantly reduced BrdU^+^/DCX^+^ double immunostaining (p = 0.0008), but SCO with SJ treatment did not protect against the loss of neuronal differentiation (Fig. 4C,D; p < 0.999). The DCX-positive neurons of FSJ-treated mice exhibited neurite elongation and branching when compared to those of SCO-treated mice (FSJ 125 mg/kg, FSJ 250 mg/kg, FSJ 500 mg/kg; p = 0.0344, p = 0.0064, p = 0.0033, respectively). These findings reveal that FSJ administration prevents the loss of neuronal differentiation with SCO treatment. In BrdU/NeuN double immunostaining experiments, newly generated BrdU-positive cells of CON-treated mice were co-labeled with NeuN in the GCL of the DG, suggesting that surviving cells differentiated to mature neurons within 14 days (arrows, Fig. 4E). Few mature BrdU/NeuN double-labeled cells were observed in SCO-treated mice, indicating that SCO interfered with the proliferation, differentiation and survival of cells in the DG of the hippocampus (arrowheads, Fig. 4E; p < 0.0001). SJ did not affect the observed SCO-induced reduction in the number of co-labeled cells (arrowheads, Fig. 4E,F; p > 0.999). Interestingly, most of the surviving BrdU-positive cells in FSJ-administered mice exhibited remarkable labeling of both BrdU and NeuN (arrows, Fig. 4E). Furthermore, quantitative analyses revealed that the reduced number of BrdU^+^/DCX^+^ or BrdU^+^/NeuN^+^ cells after SCO treatment was restored by FSJ but not SJ treatment (Fig. 4D, [F_(7,31)_ = 8.018, p < 0.0001]; Fig. 4F, [F_(7,42)_ = 8.562, p < 0.0001]).

### FSJ but not SJ attenuated SCO-induced oxidative stress, decreased ACh levels, down-regulated ChAT expression and increased AChE activity in the hippocampus

**Figure 5:**
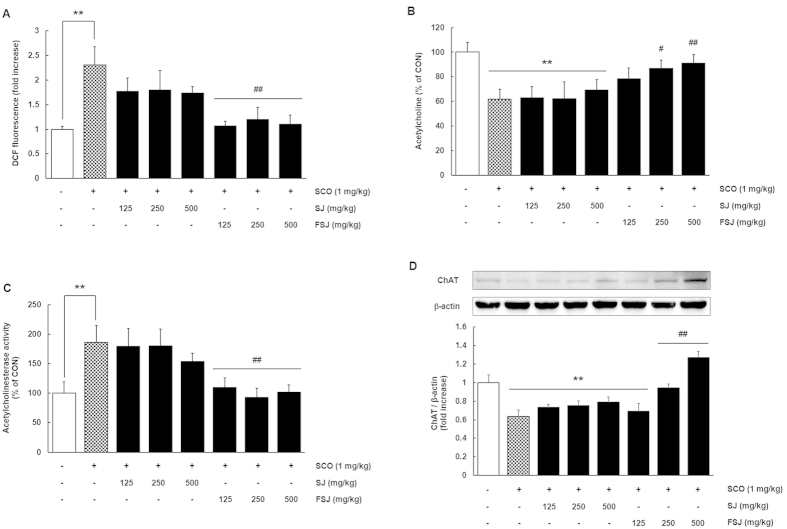
Effects of SJ and FSJ on oxidative stress, ACh levels and AChE activity in the hippocampus. (**A**) DCFDA dye was used to determine the antioxidant properties of SJ and FSJ in the hippocampus. Data are presented as the mean ± SEM (*n* = 6–8 mice/group). **p < 0.01, compared with CON-treated group; ^##^p < 0.01, compared with SCO-treated group. In (**B**,**C**) the ACh level and AChE activity in tissue homogenates of hippocampus from CON-treated, SCO-treated, SJ-treated, or FSJ-treated mice were determined using the Amplex red ACh/AChE assay kit. Data are presented as the mean ± SEM (*n* = 6–8 mice/group). **p < 0.01, compared with CON-treated group; ^#^p < 0.05, ^##^p < 0.01, compared with SCO-treated group. (**D**) The effects of SJ and FSJ on expression of ChAT in the hippocampus of mice administered SCO are shown. A representative immunoblot from one of three independent experiments shows the results for the antibodies against ChAT and β-actin. The lower graph shows the quantification of ChAT expressed as the ratio of ChAT/β-actin. Data are presented as the mean ± SEM (*n* = 6–8 mice/group). **p < 0.01, compared with CON-treated group; ^##^p < 0.01, compared with SCO-treated group.

Oxidative stress is associated with the cognitive deficits that are observed in AD, depression, and the SCO-induced amnesia mouse model^22^,^23^. To investigate the antioxidant effects of SJ and FSJ in the hippocampus, we examined reactive oxygen species (ROS) production in the hippocampus using DCFDA dye. The hippocampal ROS levels were elevated two-fold by SCO treatment (Fig. 5A; p = 0.0033), whereas FSJ completely ameliorated the increased ROS levels (FSJ 125 mg/kg, 250 mg/kg, 500 mg/kg; p = 0.0089, p = 0.0243, p = 0.0031, respectively). The SJ-treated group showed no significant differences compared with the SCO-treated group with respect to ROS levels (SJ 125 mg/kg, 250 mg/kg, 500 mg/kg; p = 0.4819, p = 0.5447, p = 0.3614, respectively, [F_(7,43)_ = 3.752, p = 0.0029]).

ACh, a major excitatory neurotransmitter in neurons, is important for the formation of long-term memories and retention of existing memories^24^. Synthesis of ACh from acetyl-CoA and choline occurs at cholinergic synapses through the action of choline acetyltransferase (ChAT) enzyme. ACh is influenced by AChE, which hydrolyzes ACh to acetate and choline in the synaptic cleft. Cognitive impairments in neurodegenerative diseases, including AD and the SCO-induced mouse model of amnesia, have been related to reductions in ACh and increased levels of AChE in the hippocampus^25^,^26^. Herein, the ACh levels were significantly lower and the activity of AChE was significantly higher in SCO-treated mice compared to CON mice (Fig. 5B,C; p = 0.0041 and p = 0.0097, respectively), supporting previous findings showing that SCO reduced the ACh levels in the brain^26^. There were no significant differences between SCO-treated and SJ-treated groups (p > 0.999); however, FSJ protected against the SCO-induced ACh decrease in the hippocampus (ACh, p < 0.05; AChE, p = 0.02). Expression of ChAT in the hippocampus was also decreased in SCO-treated mice (Fig. 5D; p = 0.0017), but FSJ effectively prevented the decreased expression of hippocampal ChAT (FSJ 250 mg/kg, p = 0.0086; FSJ 500 mg/kg, p < 0.0001). Therefore, these results indicate that FSJ has an anti-amnesic effect and protects against SCO-induced oxidative stress, reduced ACh, elevated AChE activity, and decreased ChAT in the hippocampus (ACh, [F_(7,44)_ = 2.766, p = 0.0180]; AChE, [F_(7,44)_ = 3.795, p = 0.0026]; ChAT, [F_(7,32)_ = 10.88, p < 0.0001]).

### FSJ prevented the SCO-induced suppression of BDNF, pCREB, and pAkt in the hippocampus

**Figure 6:**
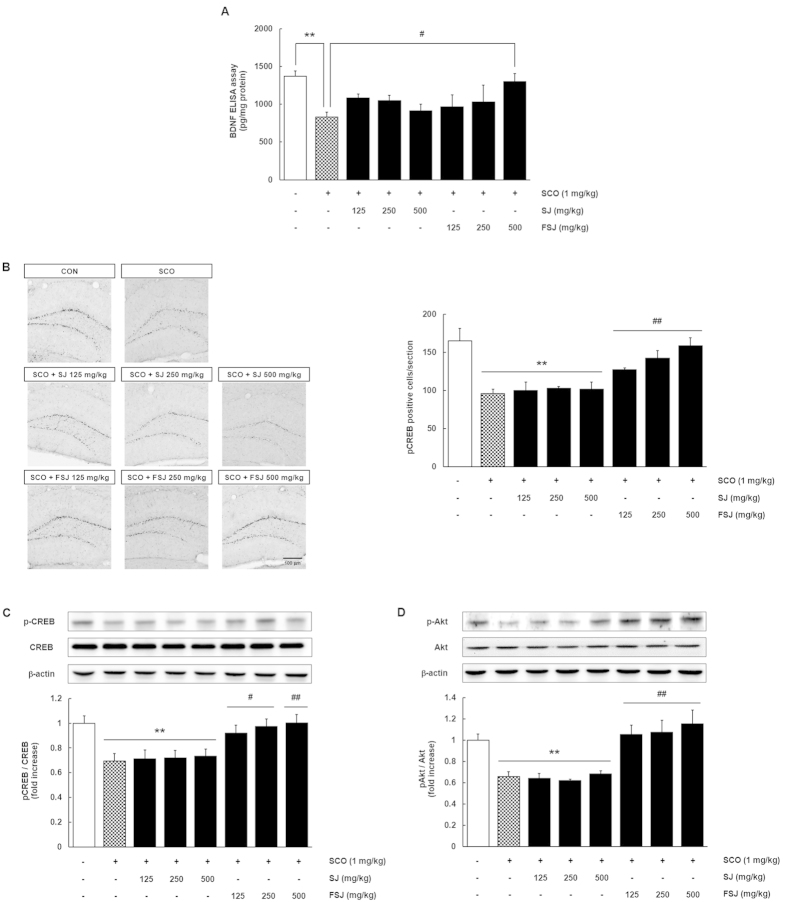
FSJ has anti-amnesic effects by preventing the SCO-induced reduction of hippocampal BDNF, CREB, and Akt. (**A**) Levels of hippocampal BDNF from each mouse group were determined quantitatively using the ELISA assay. Data are presented as the mean ± SEM (*n* = 6–8 mice/group). **p < 0.01, compared with CON-treated group; ^#^p < 0.05, compared with SCO-treated group. (**B**) The representative images show DAB immunostaining for pCREB in the hippocampal DG for each 14 day drug treatment. The scale bar = 100 μm. The graph shows the number of pCREB-positive cells in the DG. Data are presented as the mean ± SEM (n = 5 mice/group). **p < 0.01, compared with CON-treated group; ^##^p < 0.01, compared with SCO-treated group. (**C**) The effects of SJ or FSJ on pCREB expression in the hippocampus of mice administered SCO are shown. A representative immunoblot from one of three independent experiments shows the results for the antibodies against pCREB, CREB, and β-actin. The lower graph shows the quantification of pCREB expressed as the ratio of pCREB/CREB. Data are presented as the mean ± SEM (*n* = 6–8 mice/group). **p < 0.01, compared with CON-treated group; ^#^p < 0.05, ^##^p < 0.01, compared with SCO-treated group. (**D**) The effects of SJ and FSJ on pAkt expression in the hippocampus in mice treated with SCO are shown. A representative immunoblot from one of three independent experiments shows the results for the antibodies against pAkt, Akt, and β-actin. The lower graph shows the quantification of pAkt expressed as the ratio of pAkt/Akt. Data are presented as the mean ± SEM (*n* = 6–8 mice/group). **p < 0.01, compared with CON-treated group; ^##^p < 0.01, compared with SCO-treated group.

BDNF plays an important role in the development and synaptic plasticity of the brain and also plays an important role in learning and memory through CREB activation^27^,^28^. The BDNF/CREB signaling pathway is reduced in neurodegenerative diseases and depression^29^,^30^. To determine whether SCO treatment diminished the levels of BDNF or phosphorylated CREB (pCREB) and to determine whether this contributed to the neuroprotective effects of FSJ on SCO-induced hippocampal neurogenesis and cognitive impairment, we examined BDNF and pCREB levels in the hippocampus. BDNF levels in the hippocampus were dramatically reduced in mice treated with SCO for 14 days, but SJ treatment did not restore this effect (Fig. 6A; SCO, p = 0.0091; SJ, p > 0.29). In contrast, hippocampal BDNF levels were not significantly diminished in 500 mg/kg FSJ-treated mice (p = 0.0273, [F_(7,44)_ = 2.574, p = 0.0258]). Hippocampal pCREB expression was determined by immunohistochemistry staining and western blot analysis and was decreased in SCO-treated mice (Fig. 6B,C; p = 0.0089), but FSJ effectively protected against the observed diminished pCREB expression (FSJ 125 mg/kg, FSJ 250 mg/kg, FSJ 500 mg/kg; p = 0.0099, p = 0.0088, p = 0.0047, respectively; [F_(7,32)_ = 5.125, p = 0.0006]). It has been reported that BDNF increases Akt activation and that this stimulates CREB phosphorylation^28^. We examined Akt activation to investigate the molecular mechanism by which FSJ may promote hippocampal neurogenesis and facilitate cognitive function in the SCO-induced amnesia mouse model. Our results showed that FSJ, but not SJ, protected against SCO-induced Akt inactivation in the hippocampus (Fig. 6D; SCO, p = 0.0076; SJ, p = 0.9997; FSJ, p < 0.005; [F_(7,32)_ = 9.180, p < 0.0001]). These findings suggest that FSJ may promote cognitive function and hippocampal neurogenesis through a mechanism associated with the activation of neurogenic factors and Akt in the SCO-induced mouse model of amnesia.

### FSJ did not improve hippocampal neurogenesis or cognitive function

**Figure 7:**
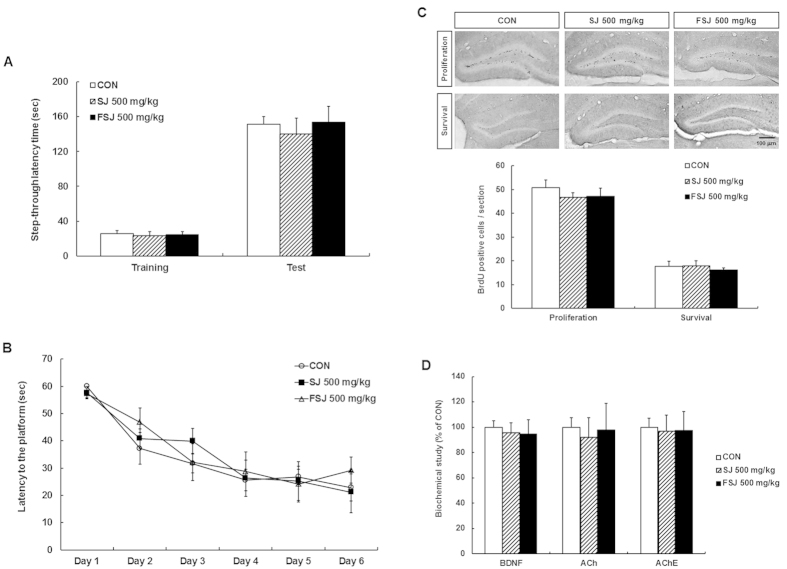
Effects of SJ and FSJ on hippocampal neurogenesis and cognitive function under normal conditions. (**A**) The cross-over latency time is shown for the passive avoidance test. (**B**) The latency time to find the hidden platform is shown for the spatial reference learning and memory test that was performed for 6 days. The values shown are means ± SEM (n = 6). (**C**) Representative images are shown that indicate proliferation or survival of newly-generated cells in the DG of the hippocampus for CON-treated, 500 mg/kg SJ-treated, or FSJ-treated mice. The scale bar = 100 μm. The graph shows the number of BrdU-positive cells in the proliferation and the survival group. Data are presented as the mean ± SEM (n = 5 mice/group). (**D**) The level of BDNF, level of ACh, and AChE activity in hippocampus tissue homogenates from CON-treated, 500 mg/kg SJ-treated, or FSJ-treated mice were determined. Data are presented as the mean ± SEM (n = 6–8 mice/group).

We next determined whether FSJ may enhance hippocampal neurogenesis and learning and memory under normal conditions. Mice were orally administered SJ or FSJ without SCO treatment for 14 days (Fig. 1). We then performed the passive avoidance test and MWM test to examine the effects of SJ and FSJ on cognitive function. Mice treated with 500 mg/kg of either SJ or FSJ did not show improvement in the step-through latency time compared to CON-treated mice (Fig. 7A; treatment effect [F_(2,35)_ = 0.8191, p = 0.4491], time effect [F_(1,35)_ = 161.3, p < 0.0001], and treatment × time interaction [F_(2,35)_ = 1.440, p = 0.2507]). In the MWM test, spatial learning and memory were also not affected by treatment with SJ or FSJ (Fig. 7B; treatment effect [F_(2,108)_ = 1.573, p = 0.2121], time effect [F_(5,108)_ = 28.90, p < 0.0001], and treatment × time interaction [F_(10,108)_ = 0.7366, p = 0.6886]). Our results also showed that neither SJ nor FSJ affected the proliferation and survival of newly-generated cells in the hippocampus (Fig. 7C; proliferation group, [F_(2,12)_ = 1.648, p = 0.2332]; survival group, [F_(2,11)_ = 1.406, p = 0.2860]). The BDNF signaling pathway and ACh/AChE activity were also unaffected by SJ and FSJ treatments (Fig. 7D; BDNF, [F_(2,18)_ = 0.8712, p = 0.4353]; ACh, [F_(2,17)_ = 0.05011, p = 0.9513]; AChE, [F_(2,18)_ = 0.1060, p = 0.9000]). These results suggest that SJ and FSJ do not have neurogenic capabilities but exert neuroprotective effects under SCO-induced amnesic conditions.

## Discussion

Collectively, the present study revealed that treatment with SCO impaired learning and memory. Multiple histological and biochemical experiments were conducted that revealed decreased hippocampal neurogenesis, a changed cholinergic system (ACh/AChE/ChAT) and an altered BDNF/CREB/Akt pathway; however, FSJ ameliorated these abnormalities and thereby protected learning and memory from the effects of SCO treatment.

Neurodegenerative diseases and aging-related dementia are characterized by cognitive dysfunction, including memory impairments and difficulties learning new information, due to a decline in normal neuronal circuit activity in the hippocampus. Data from passive avoidance and MWM tests showed that SCO-treated mice exhibited progressive learning dysfunction and memory retention deficits; however, co-treatment with FSJ not only shortened the latency time and cumulative total distance but also ameliorated swimming patterns. These findings suggest that FSJ has anti-amnesic effects in the SCO-induced mouse model of amnesia.

The cholinergic system is regarded as important in cognitive deficits such as mild cognitive impairment, dementia and early stages of AD^31^,^32^. In the cholinergic system, ACh is an important neurotransmitter that is synthesized from choline and acetyl-CoA. ACh is associated with learning and memory and is hydrolyzed by AChE to form acetate and choline in the synaptic cleft. An imbalance in the cholinergic system, such as the reduced ACh levels and excessively increased AChE activity found in the hippocampus of aging-induced dementia or AD patients, is associated with loss of cognitive function^33^,^34^. Our study showed that ACh levels and ChAT expression were reduced and AChE activity was elevated in the hippocampus during SCO treatment. Previous studies have reported that the increases in hippocampal ACh levels caused by over-activity of AChE could disrupt the cholinergic system in the brain, resulting in cognitive dysfunction^35^. However, FSJ effectively attenuated the SCO-induced dysfunction of the cholinergic system in the hippocampus, suggesting that FSJ has anti-amnesic activities that prevent learning and memory impairments by modulating the cholinergic system.

Previous studies have revealed that AChE inhibition enhances adult hippocampal neurogenesis by activating neurogenic signaling mechanisms in normal mice and in a depression mouse model^36^,^37^. Because the cholinergic system and neurogenesis occur in the hippocampus and play a pivotal role in learning and memory, an enhanced cholinergic system is correlated with hippocampal neurogenesis. In the DG of the hippocampus of adult mouse brains, newborn cells are generated from the proliferation of neural stem/progenitor cells; then newborn cells differentiate into new neurons. Newborn neurons become functionally integrated into the synaptic circuitry in the DG of the hippocampus^38^. Our data showed that SCO treatment impaired the proliferation and survival of newly-generated cells in the SGZ of the hippocampal DG; however, FSJ significantly attenuated the SCO-induced decline of newborn cells. During hippocampal neurogenesis, newborn cells of each phase and cell type are known to express specific markers^39^. In the brain, DCX is expressed in neuroblasts and immature neurons, and NeuN is expressed in mature neurons. Herein, SCO treatment significantly decreased DCX and NeuN immunostaining, whereas treatment with FSJ effectively attenuated the reduction in these immunoreactivities. These findings suggest that FSJ improves cognitive function by ameliorating SCO-induced suppression of hippocampal neurogenesis. The signaling pathway involving BDNF, CREB, and Akt is known to play an important role in the modulation of hippocampal neurogenesis and may include specific neurogenic effects such as an enriched environment, dietary restrictions, and learning^40^,^41^,^42^. Our biochemical studies have revealed that expression of hippocampal BDNF, CREB, and Akt was decreased after SCO treatment, and this may have mediated the impairment in SCO-induced hippocampal neurogenesis that was observed. Therefore, our study demonstrated that FSJ has protective and therapeutic effects in the SCO-induced mouse model of amnesia and may be associated with improvements in hippocampal neurogenesis, in the cholinergic system, and in the signaling pathway that involves BDNF, CREB, and Akt.

We investigated how FSJ modulates hippocampal neurogenesis and cognitive function as well as its molecular mechanisms in the SCO-induced mouse model of amnesia. HPLC analysis data in our previous study identified that 4 active compounds in SJ or FSJ were detected by HPLC analysis: liquiritin and liquiritigenin (*Glycyrrhizae Radix et Rhizoma*), nodakenin (*Angelicae Gigantis Radix*), and cinnamyl alcohol (*Cinnamon Bark*)^14^. Interestingly, our previous study was the first to report that fermentation of *Lactobacillus* increased the levels of liquiritigenin and cinnamyl alcohol, which has neuroprotective effects on AD transgenic mice and prevents amyloid beta-induced neuronal cell death^43^,^44^. Cinnamyl alcohol, a metabolite of cinnamaldehyde, abundantly exists in Cinnamon Bark and reportedly has inhibitory effects on AChE activity and cancer cell growth^45^. In addition, cinnamaldehyde and Cinnamon Bark extract can protect against neurodegenerative diseases, including AD and dementia, by preventing inflammation and suppressing neurofibrillary tangle formation^46^,^47^. This evidence supports the hypothesis that an increase in active compounds *via* the fermentation of SJ might contribute to the hippocampal neurogenesis and cognitive function in the SCO-induced mouse model of amnesia. Therefore, our present study demonstrates that FSJ promotes hippocampal neurogenesis and cognitive function in the SCO-induced mouse model of amnesia. Fermentation of SJ with *Lactobacillus* is thought to elevate its bioavailability and to improve its absorption. A previous study found that the active compounds of the individual herbs in SJ, including ginsenoside Rb1 (*Ginseng Radix Alba*), Atractylenolide III (*Atractylodis Rhizoma Alba*), Ergosterol (*Hoelen*), Paeoniflorin (*Paeoniae Radix*), Ligustilide (*Cnidii Rhizoma*), Betaine (*Astragali Radix*), Gingerone (*Zingiberis Rhizoma Crudus*), Sanjoinine A (*Zizyphi Fructus*), and 5-Hydroxymethylfurfural (*Rehmanniae Radix Preparata*), had potent beneficial effects when used in various animal models, including SCO-induced amnesia mice, AD transgenic mice, D-galactose-induced aging mice, and Parkinson’s disease (PD) model mice^48^,^49^,^50^,^51^,^52^,^53^,^54^,^55^. Our future studies should evaluate the active compounds of SJ and FSJ using HPLC analysis.

In the present study, we demonstrate that for SCO-induced amnesia in a mouse model, FSJ has potent anti-amnesic effects that may be mediated by improving hippocampal neurogenesis and cognitive functioning, restoring the hippocampal cholinergic system, and up-regulating the hippocampal pathway involving BDNF, CREB, and Akt signaling. Our observations provide the first evidence that FSJ may be a promising therapeutic drug for the treatment of amnesia and aging-/neurodegenerative disease-related cognitive impairment.

## Methods

### Reagents and antibodies

Scopolamine hydrobromide, 5′-Bromo-2′-deoxyuridine (BrdU), and 4% parafor-maldehyde (PFA) solution were purchased from Sigma-Aldrich Co. (St. Louis, MO, USA). 2′-7′-Dichloro-fluorescin diacetate (DCFDA) was purchased from Invitrogen Molecular Probes (Eugene, Oregon, USA). Triton X-100 and 3% goat serum were purchased from Biobasic (Biobasic Inc., Ontario, Canada) and Gibco (Gibco BRL, Grand Island, NY, USA), respectively. Staining for immunohistochemistry, primary antibodies against BrdU, doublecortin (DCX) and neuronal nuclei (NeuN) were obtained from Abcam (Abcam, Cambridge, MA, USA), Cell Signaling Technology (Danvers, MA, USA)and Millipore (Millipore, Billerica, MA, USA), respectively. Anti-phosphorylated (p) Akt (ser 473), total Akt, pCREB (ser 133), total CREB were purchased from Cell Signaling Technology. Anti-β-actin was obtained from Santa Cruz Biotechnology Inc. (Santa Cruz, CA, USA). Secondary antibodies for immunostaining were purchased from Vector Laboratories (Vector Laboratories Inc., CA, USA) or Molecular Probes (Eugene, OR, USA). Secondary antibodies for Western blotting were obtained from Thermo Scientific (Thermo Scientific, MA, USA).

### Animals

Male C57BL/6 mice (4 weeks old) were purchased from Samtako Bio Korea (Osan, Korea) and maintained under temperature- and light-controlled conditions (20–23 °C, 12 h light/12 h dark cycle) with food and water provided *ad libitum*. All animals were acclimatized for 7 days prior to drug administration. The institutional animal care committee of Korea Institute of Oriental Medicine approved the experimental protocol (#14–077) and performed according to the guidelines of the Animal Care and Use Committee at KIOM.

### Drug administration

The doses of SJ and FSJ used for experiments was determined based on the data from preliminary *in vitro* experiments. The *in vitro* data indicated that 500 μg/ml SJ, autoclaved SJ (ASJ), and FSJ had protective effects against hydrogen peroxide or glutamate-induced cell loss in SH-SY5Y cells or primary cultured cortical neurons, respectively (see Supplementary Fig. S2). SJ, ASJ, and FSJ all exhibited anti-oxidative activity in SH-SY5Y cells (see Supplementary Fig. S2). However, the neuroprotective effects of FSJ were more pronounced than those of SJ and ASJ at the 500 μg/ml dose. Thus, considering the *in vitro* data, we decided to administer 125, 250, and 500 mg/kg doses of SJ and FSJ to mice. A total of 167 male C57BL/6 mice were used in this study and were divided into ten groups according to body weight: (1) CON (vehicle-control); (2) SCO (1 mg/kg); (3) SCO + SJ 125 mg/kg; (4) SCO + SJ 250 mg/kg; (5) SCO + SJ 500 mg/kg; (6) SCO + FSJ 125 mg/kg; (7) SCO + FSJ 250 mg/kg; and (8) SCO + FSJ 500 mg/kg; (9) SJ 500 mg/kg; and (10) FSJ 500 mg/kg. Additionally, using 5–8 mice per group per study, mice of each group were divided to be used for three studies: (1) a proliferation study; (2) a survival study; and (3) a behavior + biochemical study. SCO was injected intraperitoneally (i.p.) once a day for 14 days and then for an additional 7 days during behavioral analyses. SJ or FSJ were administered orally for the same 14 days (2 h after every SCO injection). SCO, SJ and FSJ were dissolved in 0.9% physiological saline, and mice in the CON and SCO groups were administered an equal volume of 0.9% physiological saline. To evaluate new cell generation (in the proliferation study), mice in each group were administered six intraperitoneal injections of BrdU, a marker of proliferative cells in the S-phase, (50 mg/kg twice daily for 3 days) on the last three days of CON, SCO, SJ, or FSJ administration. To evaluate new cell survival (in the survival study), mice were administered BrdU for three consecutive days prior to CON, SCO, SJ, or FSJ administration. The *in vivo* experimental design is summarized in Fig. 1.

### Preparation of Sipjeondaebotang (SJ) and fermented SJ (FSJ)

The 12 medical herbs were used in this study (see Supplementary Table. S1). The medical herbs were purchased from the local vendor Hyundai Herbal Market (Yeongcheon, Korea) and deposited in the herb bank of KM-Application Center, Korea Institute of Oriental Medicine (KIOM; Daegu, Korea) after verifying by Professor Ki Hwan Bae of the College of Pharmacy, Chungnam National University (Daejeon, Korea). Water extract of SJ was extracted in distilled water (24,945 ml) at 115 °C in shaking incubator for 3 h. SJ solution was filtered through filter paper (Whatman filter paper #1) and then the filtrate was lyophilized (yield; 29.53%). The SJ was used to prepare FSJ by the addition of 249.45 ml of *Lactobacillus* (1–5 × 10^8^ colony-forming unit (CFU)/ml). Pure culture of *Lactobacillus* was obtained from Korea Food Research Institute (KFRI, Sungnam, Korea). Before experimental use, the bacterial strain was incubated in 50 ml of MRS broth (Difco^TM^ Lactobacilli MRS Broth, Becton Dickinson, Franklin Lakes, NJ, USA) at 37 °C for overnight. The SJ was fermented with *Lactobacillus* at 37 °C for 48 h and then filtered with a 60 μm nylon net filter (Millipore), precipitated overnight, lyophilized and stored in desiccator at 4 °C (yield; 28.06%). The freeze-dried extract powder was freshly dissolved in 0.9% physiological saline before use.

### Tissue preparation

For biochemical analyses, the tissues were homogenized in RIPA buffer (Millipore), protease inhibitor cocktail and phosphatase inhibitor cocktail (Roche, Mannheim, Germany) and centrifuged (12,000 rpm for 15 min at 4 °C). The supernatant was stored at −80 °C until required for biochemical analysis. For histological analyses, mice were anesthetized and perfused intracardially with 4% paraformaldehyde (PFA) in 0.1 M PBS (pH 7.4). After fixative perfusion, brains were removed, placed in the same fixative at 4 °C for overnight, and transferred to a 30% sucrose solution. Cryoprotected brains were sectioned six series at 40 μm in the coronal plane using a freezing microtome (Leica Biosystems, Wetzlar, Germany). An average of 6–8 sections (total 240–320 μm thickness) including the hippocampus per brain was used for immunohistochemical staining and blindly analyzed by investigator. All the sections including the hippocampus were collected in Dulbecco’s PBS (DPBS) solution containing 0.1% sodium azide, and stored at 4 °C.

### Immunohistochemistry

Staining for newly-generated cells or neuronal differentiation in the DG was performed as reported previously^56^. Sections were imaged using an Olympus TH4–200 microscope (Olympus, Tokyo, Japan). Cells in every sixth section throughout the entire rostro-caudal region of the hippocampus were counted. BrdU-positive cells were counted within the SGZ (for the proliferation study) and the GCL (for the survival study) of the DG in the hippocampus, from the top to the bottom of the sections. The reference region consisted of the GCL of the DG. All cell counts were performed by an investigator (HRP) who was blinded to each mouse treatment group.

### Double-label immunohistochemistry

Double-label immunostaining was performed as reported previously^56^. Confocal z-stack images were acquired using FV10i FLUOVIEW Confocal Scanning Microscope (Olympus, Tokyo, Japan).

### Western blot analysis

Protein concentrations were determined using bicinchoninic acid (BCA) assay kit (Thermo Scientific, MA, USA) with bovine serum albumin standard. Samples (30 μg protein per lane) were separated in SDS-polyacrylamide gels and transferred electrophoretically to Immobilon-PSQ transfer membranes (Millipore), as reported previously^57^. Band intensities were measured using FluorChem^TM^ SP software (Alpha. Innotech, San Leandro, CA, USA) and normalized to β-actin or total Akt.

### Levels of ROS in the hippocampus

ROS production was measured by fluorogenic dye, DCFDA, which is oxidized by intracellular ROS. Briefly, 5 μl of hippocampi homogenates of each group was mixed with 95 μl of phosphate buffer (pH 7.4) in black 96-well plate and then treated with 125 μM DCFDA to each well. Fluorescent compound was detected by fluorescence microplate reader (SpectraMax i3, Molecular devices, CA, USA) with excitation and emission of 495 nm and 529 nm, respectively.

### Levels of ACh and activity of AChE in the hippocampus

Hippocampal ACh levels and AChE activity were determined using commercially available Amplex red ACh/AChE assay kit (Invitrogen Molecular Probes) according to the manufacturer’s protocol. Fluorescent compound was detected by fluorescence microplate reader (SpectraMax i3) with excitation and emission of 571 nm and 590 nm, respectively.

### BDNF ELISA assay

BDNF protein levels were quantified using commercially available kit (Millipore) according to the manufacturer’s protocol. Absorbance was measured at 450 nm using a plate reader (SpectraMax i3).

### Morris water maze (MWM) test analysis

The MWM procedure was performed as described, as reported previously^56^. The MWM test was analyzed using the SMART V3.0 video tracking system (Panlab, Barcelona, Spain). SCO was injected i.p. to mice 30 min before the MWM test analysis.

### Passive avoidance test

The passive avoidance test was measured by using a shuttle box avoidance system (Med Associates Inc., St. Albans, VT, USA). Passive avoidance learning and memory was examined using the step-through latency test that takes advantage of the natural preference that mice show for a dark environment. The apparatus consists of illuminated (bright) and dark compartments. During the training day, mice were placed in the bright compartment and received an electric foot shock (0.3 mA, 2 sec) upon entering the dark compartment. On test day (1 day after training), mice were placed in the bright compartment for a maximum of 180 sec and the time taken to enter the dark compartment (the step-through latency) was measured as an indication of memory retention. No shock was given on test day. Memory performance was measured as the latency of each animal to cross over to the dark compartment. On test days, mice received intraperitoneal injections of SCO 30 min before each passive avoidance test.

### Statistical analysis

The behavior studies were evaluated with two-way analysis of the variance (ANOVA) followed by Bonferroni post-test. Other data were evaluated with one-way ANOVA by Dunnett’s test. The analyses were performed using GraphPad PRISM software^®^ (GraphPad PRISM software Inc., Version 5.02, CA, USA). Results are expressed as means ± standard error of the mean (SEM), and p-values of < 0.05 were considered as significant.

## Additional Information

**How to cite this article**: Park, H. R. *et al.* Fermented Sipjeondaebo-tang Alleviates Memory Deficits and Loss of Hippocampal Neurogenesis in Scopolamine-induced Amnesia in Mice. *Sci. Rep.* **6**, 22405; doi: 10.1038/srep22405 (2016).

## Supplementary Material

Supplementary Information
