# Supplementary material for: Fermented Sipjeondaebo-tang Alleviates Memory Deficits and Loss of Hippocampal Neurogenesis in Scopolamine-induced Amnesia in Mice
Source: Sci Rep. 2016 Mar 4;6:22405. doi: 10.1038/srep22405 (PMC4778044; doi:10.1038/srep22405)

Supplementary Information

Fermented Sipjeondaebo-tang Alleviates Memory Deficits and Loss of Hippocampal Neurogenesis in Scopolamine-induced Amnesia in Mouse

Park Hee Ra, Heeeun Lee, Hwayong Park, Won-Kyung Cho, and Jin Yeul Ma

Korean Medicine (KM)-Application Center, Korea Institute of Oriental Medicine (KIOM), Daegu, Republic of Korea

Corresponding author: Jin Yeul Ma, Korean Medicine (KM)-Application Center, Korea Institute of Oriental Medicine (KIOM), 70 Cheomdan-ro, Dong-gu, Daegu, 701–300, Republic of Korea

Phone: 82-53-940-3811; Fax: 82-53-940-3899; E-mail: jyma@kiom.re.kr

Supplementary Table S1. Crude components of sipjeondaebo-tang

Latin name	Scientific name	Amounts (g)	Source
Ginseng Radix Alba	*Panax ginseng C.A.Meyer*	240.0	Korea
Atractylodis Rhizoma Alba	*Atractylodes ovate*	240.0	Korea
Hoelen	*Poria cocos Wolf*	240.0	Korea
Glycyrrhizae Radix et Rhizoma	*Glycyrrhiza uralensis Fisch*	240.0	Korea
Rehmanniae Radix Preparata	*Rehmannia glutinosa Libosch. ex Steud*	240.0	Korea
Paeoniae Radix	*Paeonia lactiflora Pall*	240.0	Korea
Cnidii Rhizoma	*Cnidium officinale Makino*	240.0	Korea
Angelicae Gigantis Radix	*Angelica gigas N.*	240.0	Korea
Astragali Radix	*Astragalus membranaceus Bunge*	200.0	Korea
Cinnamon Bark	*Cinnamomum aromaticum Nees*	200.0	Korea
Zingiberis Rhizoma Crudus	*Zingiber officinale Roscoe*	74.5	Korea
Zizyphi Fructus	*Zizyphus jujuba Miller*	100.0	Korea
Total amounts		2494.5	

Supplementary Figure Legends

Supplemental Figure S1. Neuroprotective effects of FSJ against H_2O_2- or glutamate-induced cell loss in SH-SY5Y cells or primary cultured cortical neurons. Neuroprotective effects of FSJ against H_2O_2- or glutamate-induced cytotoxicity in SH-SY5Y cells (A) or primary cultured cortical neurons (B). Cells were pretreated with SJ, ASJ, or FSJ for 6 h, and then co-treated with 100 μM H_2O_2 or 400 μM glutamate for 24 h. The values shown are means ± SE (n = 8). **$p<0.01$, compared with vehicle without H_2O_2 or glutamate, ##$p<0.01$, compared with vehicle with H_2O_2 or glutamate, †$p<0.05$, ††$p<0.01$, compared with SJ and ASJ (C) Total intracellular ROS levels were measured using the DCFDA method. SH-SY5Y cells were exposed to SJ, ASJ, or FSJ pretreatment for 6 h, and then labeled with 50 μM DCFDA for 30 min. Cells were then treated with 100 μM H_2O_2 and analyzed immediately using a fluorescent plate reader. The values shown are means ± SE (n = 8). **$p<0.01$, compared with vehicle without H_2O_2, ##$p<0.01$, compared with vehicle with H_2O_2

Supplemental Figure S2. Measurement of body weight and organ weight in experimental mice for 2 weeks. (A) 5-week-old C57BL/6 male mice were administered CON (same volume of saline), SCO, SJ, or FSJ for 14 days. In each group, the body weight of mice increased normally, and no significant difference was observed. The values shown are means ± SE (n = 16–19). (B and C) To measure the safety of oral administration of SJ or FSJ, each group of C57BL/6 male mice were administered with 500 mg/kg SJ, FSJ, or same volume of saline for 14 days. Body weight and weight of

organs such as heart, lung, liver, kidney, and spleen were measured. The values shown are means ± SE ($n = 6$).

Supplemental Figure S1.

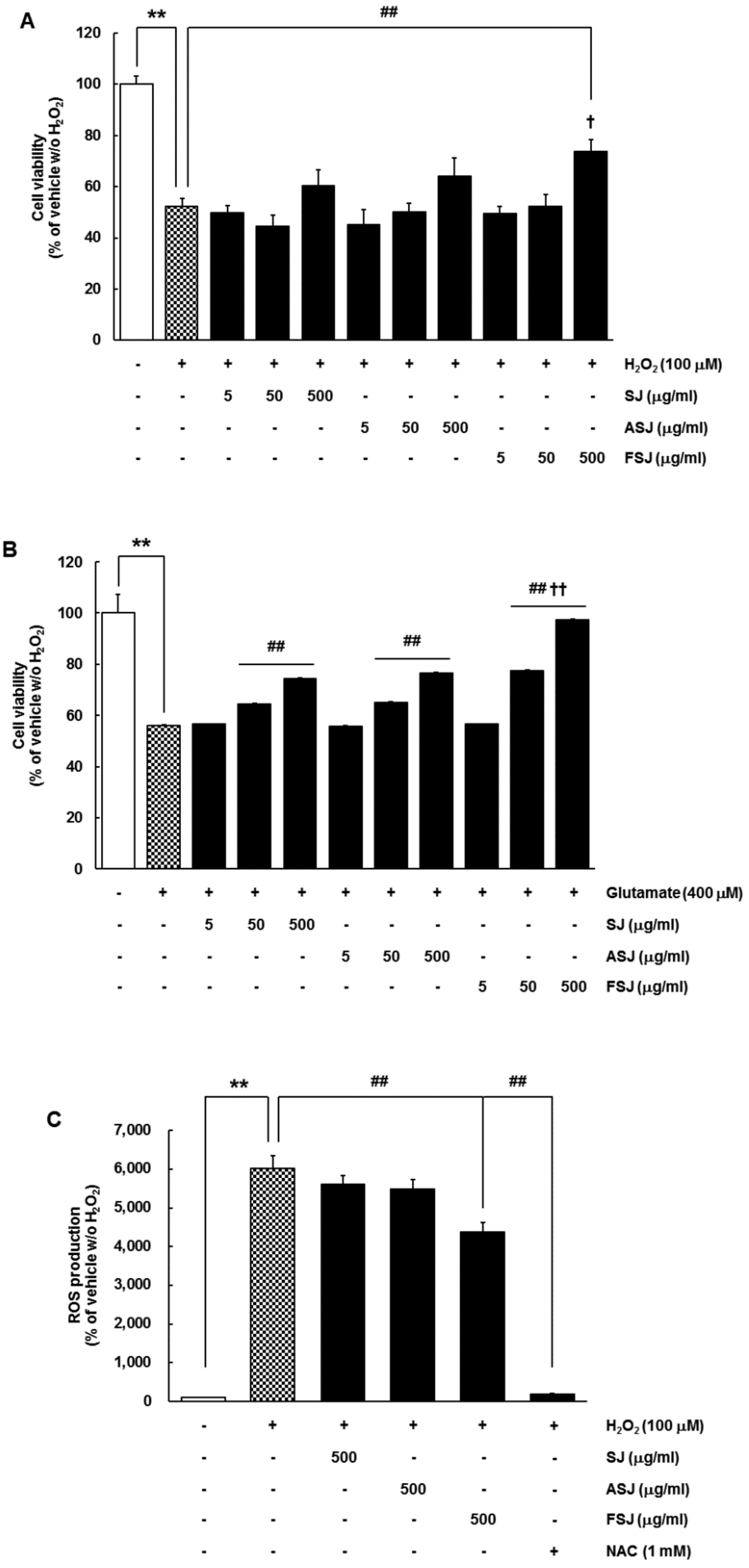

Supplemental Figure S2.

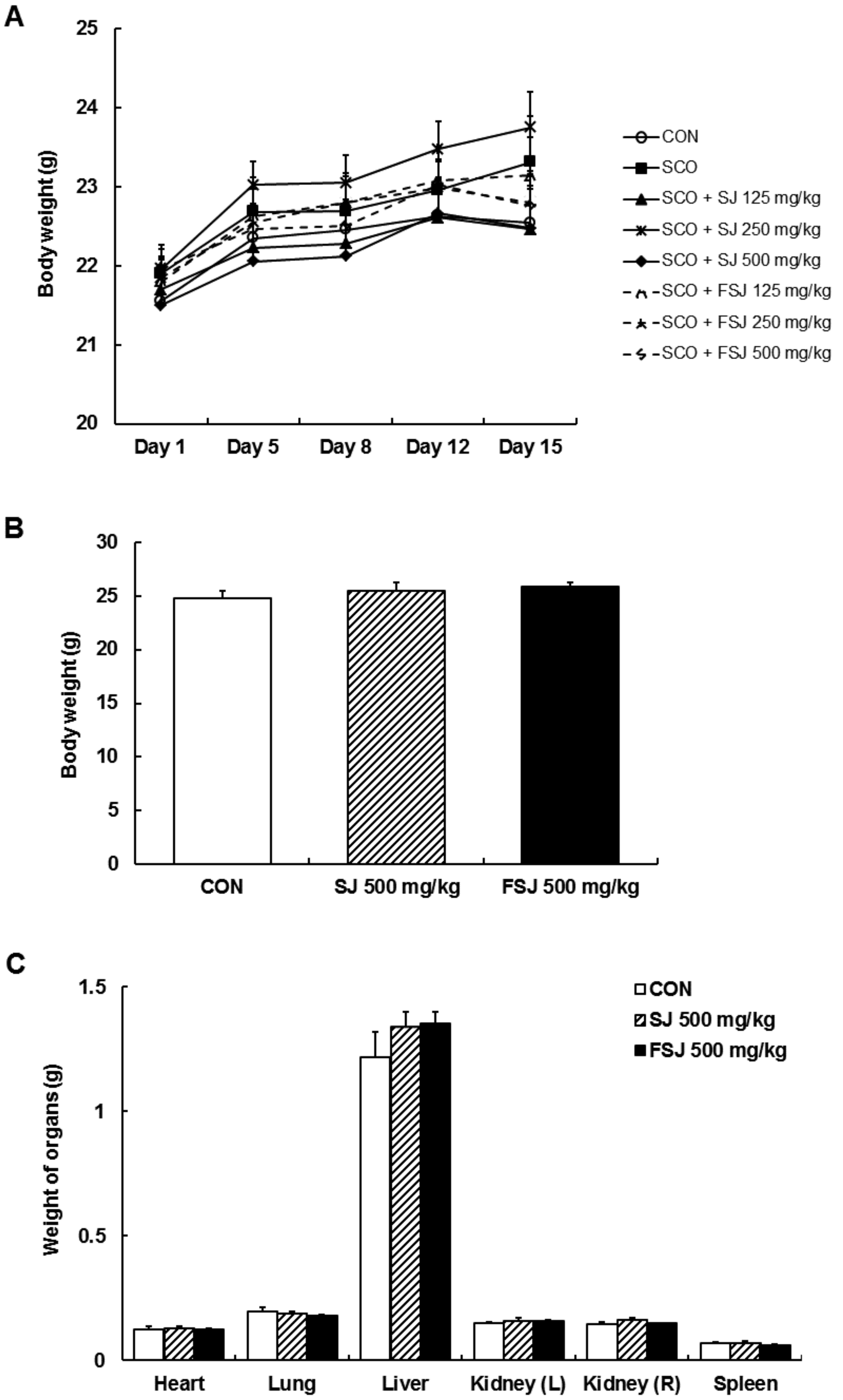

Supplement: Supplementary Information [file srep22405-s1.pdf]
